# Baicalin promotes the bacteriostatic activity of lysozyme on S. aureus in mammary glands and neutrophilic granulocytes in mice

**DOI:** 10.18632/oncotarget.15193

**Published:** 2017-02-08

**Authors:** Xuejiao Gao, Mengyao Guo, Zecai Zhang, Peng Shen, Zhengtao Yang, Naisheng Zhang

**Affiliations:** ^1^ Department of Clinical Veterinary Medicine, College of Veterinary Medicine, Jilin University, Changchun, Jilin Province 130062, People's Republic of China; ^2^ Department of Clinical Veterinary Medicine, College of Veterinary Medicine, Huazhong Agricultural University, Wuhan 430070, People's Republic of China

**Keywords:** lysozyme, baicalin, S. aureus, bacteriostasis, mastitis

## Abstract

Staphylococcus aureus causes mastitis as a result of community-acquired or nosocomial infections. Lysozyme (LYSO) is an enzyme that is upregulated in many organisms during the innate immune response against infection by bacterial pathogens. Baicalin is a bioactive flavonoid that can bind to enzymes, often to potentiate their effect. Here we tested the effects of baicalin on the activity of LYSO using the *S. aureus* mastitis mouse model and neutrophilic granulocyte model of *S. aureus* infection. In our experiments, *S. aureus* counts decreased with increasing baicalin concentration. Furthermore, qPCR and western blot analyses showed that LYSO expression was unaffected by baicalin, while fluorescence quenching and UV fluorescence spectral analyses showed that baicalin binds to LYSO. To test whether this binding increased LYSO activity, we assessed LYSO-induced bacteriostasis in the presence of baicalin. Our results showed that LYSO-induced *S. aureus* bacteriostasis increased with increasing concentrations of baicalin, and that baicalin binding to LYSO synergistically increased the antibacterial activity of LYSO. These results demonstrate that baicalin enhances LYSO-induced bacteriostasis during the innate immune response to *S. aureus*. They suggest baicalin is a potentially useful therapeutic agent for the treatment of bacterial infections.

## INTRODUCTION

*Staphylococcus aureus*, a major gram-positive pathogen, is classified as an opportunistic bacterial pathogen, which infects a diverse array of hosts [1]. *S. aureus* can be spread both through community-acquired and nosocomial infections [2]. Mastitis, which occurs most commonly by pathogenic infection during the postpartum period, poses a serious problem for humans [3] and other animals [4]. *S. aureus* is a common infectious pathogen that causes mastitis in both humans and animals [5]. *S. aureus* can infect mammary glands, resulting in serious long-term injury to mammary gland cells and ultimately damaging the structure and lactation function of the breast [6]. Until recently, there was no efficacious treatment for *S. aureus*-induced mastitis in either humans or animals. Further research is still needed to improved prophylactic and therapeutic approaches to combat *S. aureus*-induced mastitis.

Local innate immunity helps to initiate and coordinate homeostatic and defense responses in mammary glands. Lysozyme (LYSO) is a major component of the innate immune system that helps fight against infection by bacterial pathogens and viruses. Indeed, LYSO contributes to surveillance of mammalian cells membranes, fortification of the phagocytic activity of neutrophils and macrophages [7], and secretion by polymorphonuclear leukocytes [8]. LYSO breaks bacterial cell walls via hydrolysis of peptidoglycan [9] and is thus particularly effective against Gram-positive bacteria. It is extensively used in the pharmaceutical and food industries due to its anti-inflammatory, anti-viral, immune modulatory, anti-histaminic and anti-tumor effects [10].

**Figure 1:**
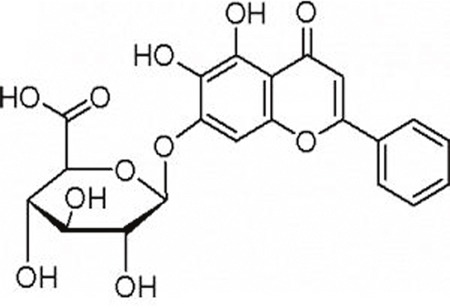
Chemical structure of baicalin.

Baicalin (Figure 1), a bioactive flavonoid isolated from the root of *scutellaria baicalensis*, exerts multiple biological functions [11–14]. Recent, studies have reported that baicalin regulates Toll-like receptors (TLRs) [15], NOD-like receptors (NODR) and other immune receptors in the innate and adaptive systems [16]. Previous studies have also suggested that LYSO can enhance the activity of some antibacterial drugs [17]. Here, we studied the effect of baicalin on the activity of LYSO in the progression of the innate immune response against infection by *S. aureus* pathogens.

## RESULTS

### *S. aureus* quantity in mammary gland tissues and neutrophil

The effect of baicalin on *S. aureus* quantity was measured by colony counting. Initially, the MIC of baicalin with *S. aureus* was confirmed at 1024 μg/ml. The concentration of baicalin was below 1024 μg/ml for treatment on tissues and neutrophils. There was no *S. aureus* in the control group in either the mammary gland tissues or neutrophils. The *S. aureus* counts were significantly increased in the MG of tissues and 0 μg/ml baicalin of neutrophils. Baicalin treatment significantly inhibited *S. aureus* growth in tissues and neutrophils in a dose dependent manner (Table 1).

Table 1Effect of baicalin on count of *S. aureus* in mammary gland and neutrophils

**Table:** 

Mammary tissues	CG	*S. aureus*	25 μg/g	50 μg/g	100 μg/g
(CFU/g)	0	2.2×10^11^	2.7×10^9^	2.2×10^7^	2.2×10^5^

**Table:** 

neutrophils	CG	*S. aureus*	25 μg/ml	50 μg/ml	100 μg/ml
(CFU/mL)	0	2.2×10^8^	1.2×10^6^	2.4×10^5^	1.3×10^4^

### Effect of baicalin on the expression of LYSO

**Figure 2:**
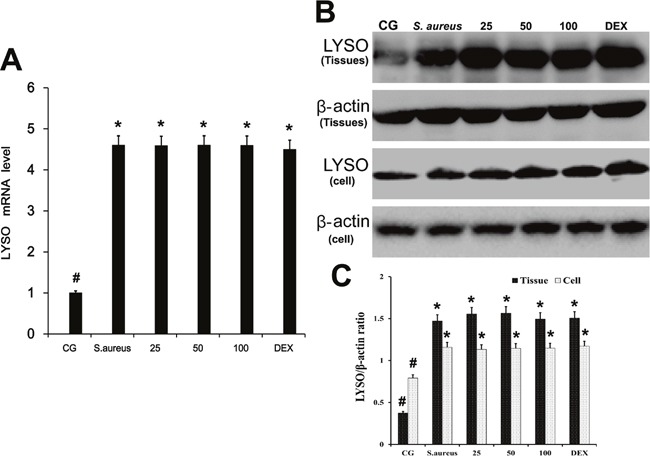
Effect of baicalin on the expression of LYSO **A**. LYSO mRNA levels in mammary tissue. **B**. LYSO protein levels. **C**. LYSO/β-actin ratio analysis. CG is the control group, *S. aureus* is the microbionation group without baicalin treatment, and the baicalin administration group consists of, 25 mg/kg, 50 mg/kg and 100 mg/kg in the animals and 25 μg/ml, 50 μg/ml and 100 μg/ml in cells. β-actin served as the control. *p < 0.05 indicates a significant difference from the CG; #p<0.05 indicates a significant difference from S. aureus.

Lysozyme is the major component of the innate immune system against infection by bacterial pathogens, particularly Gram-positive bacteria [18]. We analyzed the effect of baicalin on LYSO expression using qPCR and western blotting (Figure 2). Compared with controls, LYSO mRNA and protein levels were increased by *S. aureus* infection in mammary tissues and neutrophils. Baicalin showed no effect on the expression of LYSO. LYSO mRNA and protein levels remained high in the presence of *S. aureus* infection without baicalin treatment. Varying baicalin concentrations did not affect LYSO levels either.

### Binding of baicalin and LYSO

**Figure 3:**
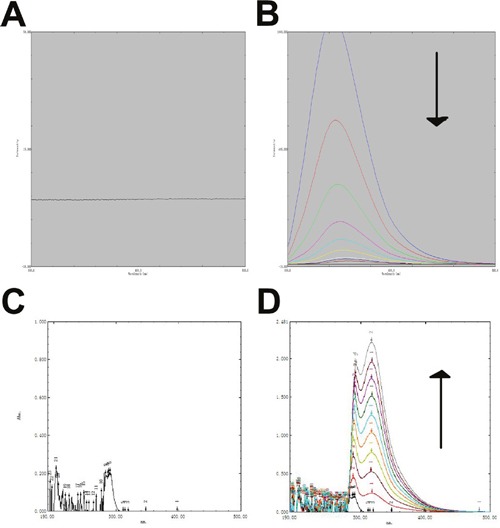
Spectroscopy experiment on the binding of baicalin and LYSO **A**. Fluorescence spectroscopy of baicalin. **B**. Fluorescence spectroscopy of baicalin at different concentrations with LYSO. **C**. UV Fluorescence spectroscopy of LYSO. **D**. The effect of baicalin on LYSO with UV Fluorescence spectroscopy. The arrows indicate the concentrations of baicalin in increasing order (0, 10, 20, 30, 40, 50, 60, 70, 80 μg/ml).

We used spectroscopy to measure potential interactions between baicalin and LYSO (Figure 3). Baicalin displayed fluorescence quenching with LYSO. The emission peak of LYSO moved from 337 nm to 294 nm. Fluorescence spectroscopy experiments showed that the fluorescence intensity of LYSO was reduced by baicalin in a dose-dependent manner (Figure 3B). We also measured UV fluorescence, which ranged between 200 and 500 nm in wavelength. These results showed that the UV fluorescence spectroscopy of LYSO was also altered by baicalin in a dose-dependent manner (Figure 3D).

### Baicalin promotes the activity of LYSO

**Figure 4:**
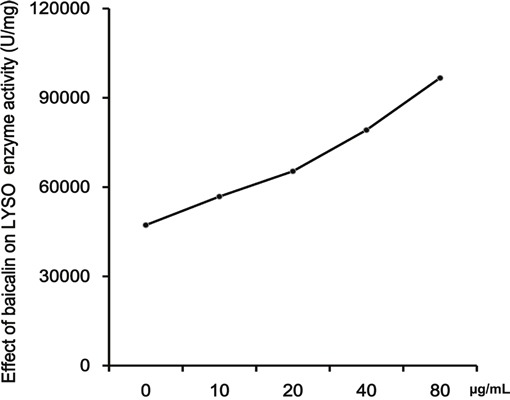
Effects of baicalin on the activity of LYSO The coactivation of baicalin and LYSO was confirmed. Effects of baicalin on the activity of LYSO were analyzed at increasing concentrations of baicalin (0, 10, 20, 40, 80 μg/ml).

Micrococcus lysoleikticus is typically used to measure the activity of LYSO by nephelometry. We used this method and our results confirmed the coactivation of baicalin and LYSO. The LYSO enzymatic activity value in the absence of baicalin was 47217.6 U/mg. The presence of baicalin enhanced LYSO activity in a dose-dependent manner (Figure 4).

### Baicalin enhances the bacteriostatic effect of LYSO on *S. aureus*

**Figure 5:**
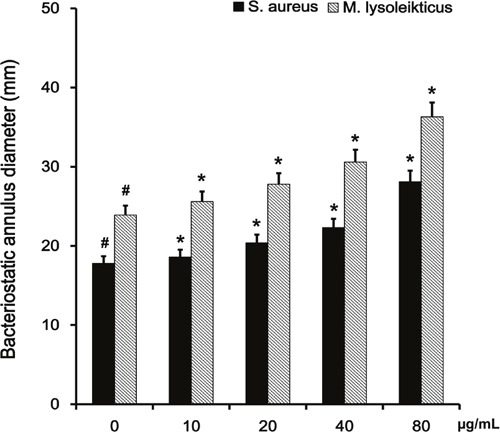
Effects of baicalin on the size of the bacteriostatic circle of LYSO The experiment to measure the antibacterial effect of LYSO on *S. aure*us with baicalin was performed *in vitro*. Micrococcus lysoleikticus was used for the case-controlled study. The concentrations of baicalin used were 0, 10, 20, 40, 80 μg/ml. *p < 0.05 indicates a significant difference from 0 μg/ml baicalin.

The size of the bacteriostatic circle is the direct method for confirming bacteriostatic action. We performed antibacterial experiments *in vitro* to measure the bacteriostatic effects of LYSO on *S. aure*us in the presence of baicalin (Figure 5). Baicalin alone did not have any bacteriostatic effects on *S. aureus* at concentrations of 10, 20, 40 and 80 μg/ml. On the other hand, LYSO exerted antibacterial effects on *S. aureus* at 100 μg/ml. Furthermore, the size of the LYSO bacteriostatic circle was increased by baicalin in a dose-dependent manner. Micrococcus lysoleikticus was used for the case-controlled study.

## DISCUSSION

*S. aureus* is the main pathogen causing mastitis in both humans and animals [3]. Mastitis, an inflammatory disease of mammary glands, is caused by infections during the postpartum period [19] and is closely related to the immune state of partial-breast [20]. An increase in the count of neutrophils is a sign of mastitis in mammary glands [21]. While polymorphonuclear leukocytes are the major cell type that control bacterial infections in mammals [22], LYSO is a major component against infection by bacterial pathogens in neutrophils [8]. Previous studies showed that baicalin treatment could significantly reduce the colony count of *S. aureus* in mammary tissues [15] and in neutrophils [1].

In the present study, the MIC of baicalin with *S. aureus* was confirmed at 1024 μg/ml, and the concentration of baicalin treatment on tissues and neutrophils was below 1024 μg/ml. Previous studies have shown that drugs can increase the expression or activity of enzymes [23]. In our study here, the expression of LYSO was not affected by baicalin in either mammary tissues or neutrophils. However, our fluorescence spectroscopy experiments on baicalin combined with lysozyme were consistent with previous studies [24]. LYSO is a small globular protein, consisting of 129 amino acid residues with six tryptophans, two tyrosines and four disulfide bonds and exhibits helical (a-helix), pleated sheet (b-sheet), turn (b-turn) and random coil secondary structure. These structural elements allow LYSO to bind drugs to aid in the treatment of some illnesses [25]. LYSO exhibits bacteriostatic activity and its interactions with some drugs (e.g., antibiotics) can have a synergistic effect [26, 27].

We used *M. lysoleikticus* (as typically done) to measure the activity of LYSO with nephelometry in the absence and presence of baicalin. LYSO breaks bacterial cell walls made of peptidoglycan via hydrolysis of the β (1–4) glucosidic bond between N-acetylmuramic acid and N-acetylglucosamine [28, 29]. *M. lysoleikticus* possesses a peptidoglycan wall [30]. Our results showed that baicalin enhanced the activity of LYSO in a dose-dependent manner. Since *S. aureus* is a Gram-positive bacterium [31], this suggests that its peptidoglycan cell wall was destroyed, resulting in apoptosis. Measuring the size of the bacteriostatic circle is the standard method to test for bacteriostatic action [32]. We analyzed the antibacterial activity of LYSO on *S. aureus* under different concentrations of baicalin. Our results showed that with increasing concentrations of baicalin, the size of the bacteriostatic circle of LYSO on *S. aureus* increased. A previous study showed that baicalin could increase the antiseptic action of acheomycin on *S. aureus*. Our results demonstrated that baicalin could also enhance the antiseptic activity of LYSO on *S. aureus*.

A previous study by our group showed that baicalin played an important role in immune regulation [15]. Local innate immunity helps to maintain homeostasis and to trigger defense responses in mammary glands [33]. LYSO is a major component in the innate immune system against infection by bacterial pathogens. In the present study, we found that baicalin inhibited the growth of the *S. aureus* by enhancing theantiseptic activity of LYSO secreted by neutrophils in mammary gland tissues. Our results here warrant further studies to firmly establish whether baicalin should be administered as a drug to treat *S. aureus* mastitis.

## MATERIALS AND METHODS

### S. aureus

*S. aureus* (ATCC 35556) was purchased from the American Type Culture Collection (ATCC. USA). *S. aureus* were resuspended in 1,000 μl PBS (2×10^8^ CFU per 30 μl). Fifty milliliters of culture aliquots were centrifuged and washed with phosphate-buffered saline (PBS) prior to resuspension. A 100-μl suspension was used per breast. The *S. aureus* suspension was injected via the teat canal of breast to induce infection in the mammary gland.

### MIC of baicalin

Baicalin (purity >99.9 %) was purchased from Sigma Chemical Company. The minimal inhibitory concentration (MIC) of baicalin with *S. aureus* was detected using the broth micro dilution method, according to the American Institute of Standardization of Clinical Laboratory (CLSI) guidelines. Baicalin was diluted multiple times with bouillon culture medium in 96-microwells plates. The initial concentration was 4096 μg/ml. Next, 100 μl of bacterium suspension were added to each well. The reaction mixture was incubated for 24 h at 37°C. The value was evaluated by measuring the change in absorbance at 600 nm using a 96-well plate reader. The percentage of growth (test/*S. aureus*) was shown as the MIC.

### Experimental animal and drug administration

Sixty adult female BALB/c mice (6-8 w old, weighing 25-28 g) were used in the present study and were provided by the Center of Experimental Animals of Baiqiuen Medical College of Jilin University in China. The procedures were performed according to the NIH Guidelines for the care and use of Laboratory Animals and were approved by the Institutional Animal Care and Use Committee of Jilin University. Baicalin was dissolved in physiological saline and intraperitoneally injected three times at 6 h, 12 h and 24 h after the microbionation in the mammary gland. Mice were postpartum in lactation period and divided into three groups as follows: 1) Microbionation group (MG), the mouse model of *S. aureus* mastitis without drug treatment; 2) baicalin administration group, which was subjected to mastitis by *S. aureus* and intraperitoneally administered baicalin at 25 mg/kg, 50 mg/kg, or 100 mg/kg, as previously described [15]; 3) control group (CG), in which mice were treated with normal saline as the vehicle control at the same volume and time point as baicalin for the baicalin administration group. *S. aureus* culture broth was centrifuged and washed with phosphate-buffered saline (PBS). Then, *S. aureus* were resuspended in PBS (1 × 10^7^ CFU per 10 μl). A 100-μl suspension was administered per breast. The *S. aureus* suspension was injected via teat canal to induce a mammary gland infection. The mammary gland tissues were weighed and homogenized with phosphate buffered saline (w/v: 1/9) on ice, and then coated bacteria underwent colony counting, while others were centrifuged at 2,000 × *g* for 40 min at 4°C. The supernatant was collected for LYSO detection.

### Neutrophil granulocyte cell culture and drug treatment

Neutrophil granulocytes were separated from fractions of heart blood obtained from mice using a Percoll separation medium density gradient. The purified neutrophils were seeded at a concentration of 3×10^7^ cells/well into 6-well plates and cultured in RPMI1640 medium supplemented with 10% fetal bovine serum in a humidified atmosphere of 5% CO_2_. After 2 h, different concentrations of baicalin were added to the wells of 6-well plates and incubated for 1 h. The concentrations were 0, 10, 20, 40 and 80μg/ml. The last well was the control with neither drug nor bacteria. Infection was induced using *S. aureus*. The viability of the bacteria was routinely confirmed by counting the number of colonies to determine the CFU/ml. Bactericidal antibacterial assays were carried out for 2 h at 37°C at a multiplicity of infection of 1:5. Then, the dilution was plated on TBS agar, and colonies were counted.

### Quantitative real-time polymerase chain reaction

Total RNA was isolated from the tissue and PMN cell samples using the TRIzol reagent and synthesized first-strand cDNA using oligo(dT) primers and Superscript II reverse transcriptase, according to the manufacturer's instructions (Invitrogen, USA). Synthesized cDNA was diluted five times with sterile water and stored at -80°C. The Primer Premier software (PREMIER Biosoft International, USA) was used to design specific primers for LYSO and β-actin based on known sequences (Table 2). Quantitative real-time PCR was performed on an ABI PRISM 7500 Detection System (Applied Biosystems, USA). Reactions were performed in a 25-μl reaction mixture, under the following conditions: 95°C for 30 s, followed by 35 cycles of 95°C for 15 s, 63°C for 30 s and 60°C for 30 s. The results (fold changes) were expressed as 2^−ΔΔCt^. β-actin served as the reference gene.

**Table 2:** Oligonucleotide primers used for qPCR

Name	Primer sequence	Product size (bp)
LYSO	Sense: 5′- CCAGCCTCCAGTCACCAT -3′	378
	Anti-sense: 5′-TCGTTTTACTCCAATTTAT-3′	
β-actin	Sense: 5′-TAAAACGCAGCTCAGTAACAGTCGG-3′	182
	Anti-sense: 5′-TGCAATCCTGTGGCATCCATGAAAC-3′	

### Western blotting analyses

The total protein of the mammary gland tissue and PMN cell samples was extracted according to the manufacturer's recommended protocol (Invitrogen, Beijing, China). The protein concentrations were determined using the BCA Protein Assay Kit and equal amounts of protein (50 μg) for fractionation on 10% SDS polyacrylamide gels. The proteins were then transferred onto polyvinylidene difluoride membranes and blocked. The membranes were then incubated with primary antibodies (LYSO and β-actin). After several washes, the membranes were incubated with secondary antibodies. Protein expression was measured using an Enhanced Chemiluminescence Detection System. β-actin was used as a loading control.

### Fluorescence measurements

Fluorescence spectroscopy experiments were executed by Fluorescence Spectroscopy (SHIMADZU Co. Japan). Spectra were measured at two temperatures (298 k and 310 k), with an excitation wavelength at 280 nm and an emission wavelength from 300 to 500 nm. The slit widths for both the excitation and emission channels were 5 nm. The samples were placed in a 1×1 cm path length quartz cuvette (2 ml). To eliminate the inner filter effects of LYSO and baicalin, the fluorescence intensities were corrected using the equation: Fcor=Fobs10 (A1+A2)/2, where Fobs is the observed fluorescence, and A1 and A2 are the sum of the absorbance of LYSO and baicalin at the measured condition, respectively. UV fluorescence measurements were also used, and the wavelength range was between 200-500 nm.

### Antibacterial activity of LYSO correlates positively with varying baicalin concentration

*Micrococcus lysodeikticus (M. lysodeikticus)*, Gram-positive bacteria, was usually a positive result for the antibacterial activity of LYSO. We selected *M. lysodeikticus* and *S. aureus*. The two bacteria were inoculated in sterile nutrient broth at 37°C for 24 h, and they were then spread on the surface of nutrient agar discs, and seeded in 20-μl test culture containing 1×10^8^ CFU/ml. The antibacterial activity of baicalin in the presence of LYSO was assessed using the agar disc diffusion method. Whatman No. 1 filter papers (diameter 4 mm) impregnated in 100 μg/ml LYSO solution with different concentrations (0, 10, 20, 40, 80 μg/ml) of baicalin were placed on the surface of the nutrient agar. The control disk was placed with water to assess the effect of water on the pathogens. The plates were incubated at 37°C for 24 h, and antibacterial activity was measured based on the inhibitory zone. The antibacterial activity test was also assessed by the value of OD_600nm_ using UV-spectrophotometry. The two bacteria were inoculated in sterile nutrient broth at 37°C for 24 h, and then each 20-μl aliquot was spread onto 4-ml sterile nutrient broth and incubated at 37°C for 24 h.

### Data analyses

Statistical analyses were performed using the SPSS software package (ver. 13 for Windows; SPSS Inc., Chicago, IL, USA). Significance was determined using one-way ANOVA with a significance level of *p-value* < 0.05. The data were assessed using the Tukey-Kramer method for multiple comparisons. All values were expressed as the mean ± SD.
